# Impact of hypokalemia on Brugada syndrome: case report unveiling mechanisms beyond QT interval prolongation

**DOI:** 10.1186/s43044-024-00574-3

**Published:** 2024-10-22

**Authors:** Muchtar Nora Ismail Siregar, Vickry H. Wahidji

**Affiliations:** Department of Cardiology and Vascular Medicine, Faculty Medicine, Gorontalo State University,, Jalan Jend, Sudirman No.6, Dulalowo Kecamatan Kota Tengah Kota, Gorontalo, 96128 Indonesia

**Keywords:** Brugada syndrome, Hypokalemia, QT interval, Case report

## Abstract

**Background:**

Brugada syndrome (BrS) is associated with an increased risk of sudden death caused by ventricular arrhythmias. The characteristic electrocardiographic appearance of ST-segment elevation of more than 2 mm with coved-type morphology in more than 1 right precordial lead is seen. Hypokalemia is known to unmask the Brugada type-1 pattern, but its exact role and mechanisms in this context are not well understood.

**Case presentation:**

We report a case of first-time diagnosis of BrS in a 51-year-old man with hypokalemia 2.8 mmol/L. Despite the normalization of potassium levels with potassium chloride (KCL), the Brugada type-1 pattern persisted on ECG. Interestingly, the corrected QT interval was shorter during hypokalemia (QTc 390 ms) compared to when potassium levels were normal (QTc 432 ms).

**Conclusions:**

This case highlights that hypokalemia can unmask the Brugada type-1 electrocardiographic pattern, but does not alter it once unmasked. The observed shorter QT interval during hypokalemia challenges the assumption that QT prolongation is the sole mechanism by which hypokalemia influences Brugada syndrome. This underscores the need for further research into additional mechanisms by which hypokalemia might trigger ventricular arrhythmias in Brugada syndrome.

## Background

Brugada syndrome (BrS) is associated with an increased risk of sudden death caused by ventricular arrhythmias. Channelopathy due to autosomal dominant genetic mutation has a characteristic electrocardiographic appearance of ST-segment elevation of more than 2 mm with coved-type morphology in more than 1 right precordial lead [1]. Diagnosis can be made based on clinical criteria and also genomic criteria [2]. Several conditions can unmask the electrocardiographic appearance of Brugada type-1 including fever, vagal stimulation, medications, and electrolyte abnormalities [3]. It has been reported that several cases of hypokalemia triggered the Brugada electrocardiographic pattern. Several theories have also been put forward regarding the mechanism by which hypokalemia causes ventricular arrhythmia in Brugada patients. However, we report a case of BrS with hypokalemia with different characteristics.

## Case presentation

A 51-year-old male presented with the primary complaint of syncope during physical activity, which lasted less than 5 min. Shortly before the syncope, the patient experienced palpitations accompanied by cold sweat. Although he had a history of frequent palpitations, he had only experienced syncope twice before. After the syncope, there were no signs of lateralization. The patient did not report any chest pain or shortness of breath. His medical history was unremarkable for hypertension, diabetes, stroke, or coronary heart disease. Additionally, there was no known family history of sudden death without a known cause.

**Fig. 1:**
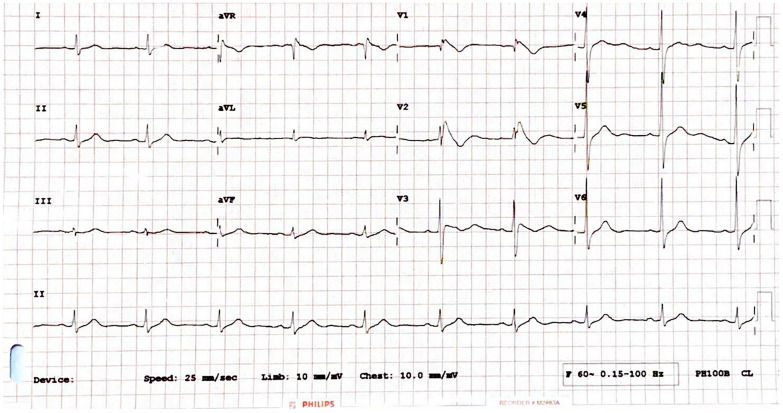
The 12 leads electrocardiography on admission showed sinus rhythm with coved ST segment elevation and inverted T waves in leads V1-V2

Physical examination in the emergency room revealed that the patient was conscious and oriented. His blood pressure was 120/80 mmHg, pulse was 78 beats per minute, breathing rate was 20 breaths per minute, and temperature was 37 °C. Other aspects of the physical examination were within normal limits. A significant laboratory finding showed a potassium level of 2.8 mmol/L. The 12-lead electrocardiography on admission revealed a right bundle branch block with coved ST-segment elevation and inverted T waves in leads V1-V2, consistent with Brugada type-1 pattern (Fig. 1). Transthoracic echocardiography did not reveal any significant abnormalities.

**Fig. 2:**
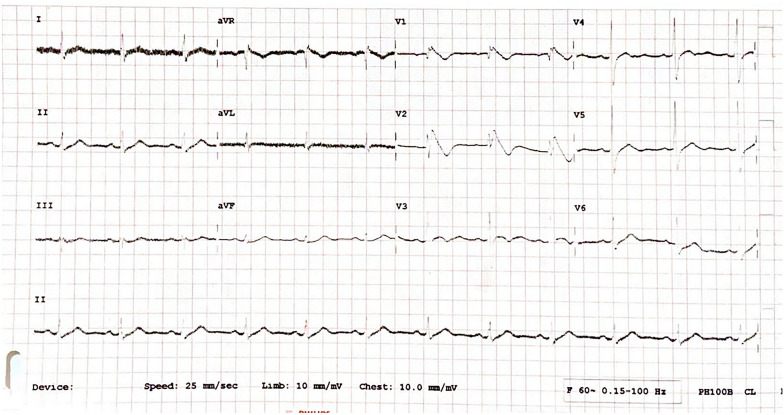
.

The patient was diagnosed with Brugada syndrome and hypokalemia. Hypokalemia was corrected by administering 25 meq of KCL solution. Potassium levels subsequently returned to normal at 4.0 mmol/L. Follow-up electrocardiography showed persistent Brugada type-1 pattern (Fig. 2). The patient was advised to be referred to a tertiary care center for implantable cardioverter-defibrillator (ICD) implantation but refused. During treatment, 12-lead electrocardiographic monitoring was performed, revealing similar results. An ambulatory ECG was also conducted to detect any malignant arrhythmias while the patient was sleeping, but no abnormalities were found. The patient is now scheduled for outpatient follow-up and screening for other family members.

We report a case of a first-time diagnosis of Brugada syndrome (BrS) in a 51-year-old man. This patient had rarely experienced syncope throughout his life, with only two recorded episodes. He was admitted to the hospital for the first time with complaints of syncope accompanied by palpitations and cold sweat. The diagnosis of BrS was made based on clinical criteria, including the electrocardiographic Brugada type-1 pattern and additional clinical criteria such as syncope, which is believed to be related to arrhythmia. Based on these criteria, the patient was a candidate for implantable cardioverter-defibrillator (ICD) implantation, but he refused the procedure. Our center also does not have access to quinidine or ablation catheter services. Therefore, we focused on educating the patient about lifestyle changes to avoid trigger factors such as drugs, alcohol, and cocaine and to manage fever [4].

Initially, we assumed that the electrocardiographic Brugada type-1 pattern was induced by electrolyte abnormalities, specifically hypokalemia. This assumption is supported by the literature indicating that the Brugada electrocardiographic pattern can be triggered by various conditions, such as fever, medications, cocaine, and electrolyte imbalances. Several case reports also describe instances where hypokalemia has triggered electrocardiographic changes characteristic of Brugada syndrome [5–8]. However, after correcting the hypokalemia and normalizing potassium levels, the electrocardiographic pattern remained unchanged, still displaying the Brugada type-1 morphology. Therefore, we concluded that the patient likely had a spontaneous Brugada type-1 electrocardiographic pattern.

**Fig. 3:**
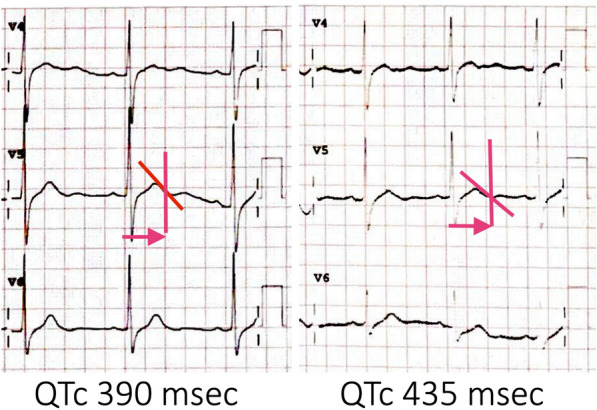
.

The next assumption we considered was whether the hypokalemia experienced by the patient could have been the cause of the syncope associated with arrhythmias. Hypokalemia is known to prolong the QT interval, which can potentially lead to ventricular arrhythmias in patients with Brugada syndrome (BrS) [9]. However, in this patient, the corrected QT interval was actually shorter during hypokalemia QTc 390 ms compared to when potassium levels were normal QTc 435 ms (Fig. 3). This discrepancy raises the question of how hypokalemia might trigger ventricular arrhythmias in Brugada syndrome if it does not align with the expected QT prolongation.

This case provides new insights into the relationship between hypokalemia and Brugada syndrome. The persistence of the Brugada type-1 pattern despite normalization of potassium levels suggests that hypokalemia's role extends beyond simple QT interval prolongation. The observation that QTc was shorter during hypokalemia challenges the conventional view that hypokalemia predominantly prolongs the QT interval. This implies that other mechanisms, potentially related to ion channel function or cardiac repolarization, may be involved in how hypokalemia influences arrhythmogenic potential in Brugada syndrome.

## Conclusions

This case underscores that hypokalemia can unmask the Brugada type-1 pattern and potentially trigger arrhythmias. The unexpected finding of a shorter QT interval during hypokalemia challenges the traditional view and suggests that the interaction between hypokalemia and Brugada syndrome involves complex mechanisms beyond simple QT interval prolongation. Further research is essential to clarify these mechanisms and improve our understanding of how electrolyte disturbances influence Brugada syndrome.

## Data Availability

All data described in this manuscript will be freely available to any scientist.

## Abbreviations

BrS: Brugada syndrome
ICD: Implantable cardioverter-defibrillator
